# Molecular characteristics of the immune escape of coronavirus PEDV under the pressure of vaccine immunity

**DOI:** 10.1128/jvi.02193-24

**Published:** 2025-04-16

**Authors:** Yunchuan Li, Shanshan Yang, Jiali Qian, Shiyu Liu, Yupeng Li, Xu Song, Qiuxia Cao, Rongli Guo, Yongxiang Zhao, Min Sun, Mi Hu, Jizong Li, Xuehan Zhang, Baochao Fan, Bin Li

**Affiliations:** 1 Institute of Veterinary Medicine, Jiangsu Academy of Agricultural Sciences, Key Laboratory of Veterinary Biological Engineering and Technology, Ministry of Agriculture, Jiangsu Key Laboratory for Food Quality and Safety-State Key Laboratory Cultivation Base of Ministry of Science and Technology, Nanjing, China; 2 Jiangsu Co-innovation Center for Prevention and Control of Important Animal Infectious Diseases and Zoonoses, Yangzhou University 38043, Yangzhou, China; 3 School of Life Sciences, Jiangsu University 12676, Zhenjiang, China; 4 School of Food and Biological Engineering, Jiangsu University 506405 , Zhenjiang, China; 5 GuoTai (Taizhou) Center of Technology Innovation for Veterinary Biologicals, Taizhou, China; Loyola University Chicago - Health Sciences Campus, Maywood, Illinois, USA

**Keywords:** coronavirus, PEDV, immune escape, immune pressure, S protein

## Abstract

Coronaviruses have undergone evolutionary changes and mutations in response to the immune pressures exerted by vaccines and environmental factors, resulting in more severe consequences during breakthrough infections. Nevertheless, the specific correlation between the evolutionary mutations of coronaviruses and immune pressures remains ambiguous. Swine coronavirus—porcine epidemic diarrhea virus (PEDV)—has existed for decades. This study utilized *in vivo* preparation of polyclonal antibodies against the PEDV and identified critical neutralizing epitopes through serial *in vitro* passaging. Then, the recombinant mutated strains were successfully constructed. *In vitro* experiments confirmed the ability of the rA1273P strain to escape neutralization by polyclonal antibodies. Both *in vitro* cell studies and *in vivo* animal experiments revealed that the strain maintains virulence and pathogenicity while evading antibody pressure post-vaccination. The pathogenicity of the strain while evading immune pressure is comparable to wild-type strains. A comparison of the S protein gene between vaccine strains and clinical strains identified mutations in 1273 amino acid positions in clinical strains. In conclusion, this study identified a novel PEDV S protein neutralizing site under immune pressure through serial passaging, indicating that the 1,273th amino acid position is prone to mutation under prolonged antibody pressure, enhancing the virus’s ability to escape hosts. This study offers new insights into the interpretation of coronavirus escape immune pressure and provides technical support for monitoring and predicting the variation and evolution of coronavirus.

**IMPORTANCE:**

Coronaviruses represent an ongoing public health threat because of high variability. Since 2010, the emergence of highly pathogenic porcine epidemic diarrhea virus (PEDV) strains has resulted in significant economic losses to the global pig industry. PEDV undergoes evolution and mutation under external immune pressure, rendering it an increasingly challenging target for prevention and control measures. Here, we prepared the polyclonal antibodies against PEDV and identified a novel neutralization epitope on the S protein (1,273th amino acids) through serial *in vitro* passaging. Furthermore, our findings indicate that the mutation of A1273P in the S protein did not alter the virulence of the PEDV but significantly enhanced its ability to escape and infect the host *in vitro* and *in vivo*. Finally, we found that the 1,273 amino acid position of the S gene has been mutated to varying degrees in clinical PEDV strains. This work provides a specific correlation between the evolutionary mutations of coronaviruses and immune pressures.

## INTRODUCTION

Coronaviruses are known to induce a range of respiratory, gastrointestinal, and central nervous system illnesses in both humans and animals, posing a significant threat to human health and resulting in substantial economic burdens (1, 2). The coronavirus belongs to the order *Nidovirales*, the family *Coronaviridae*, and the genus *Coronavirus* in the taxonomic classification. Viruses in the genus *Coronavirus* are enveloped and possess a linear single-stranded positive-sense RNA genome (3, 4). The viral genome primarily encodes four structural proteins: nucleocapsid (N), envelope (E), membrane (M), and spike (S) proteins (5). The S protein plays a crucial role in facilitating the binding of the virus envelope to host cell receptors through its receptor-binding domain and N-terminal domain, thereby mediating fusion between host cells and the virus (6). Coronaviruses exhibit a high capacity for adapting to novel environments by means of mutation and recombination, particularly through specific mutations at the S protein binding sites. This capability allows viruses to effectively alter their host range and tissue tropism, while significantly influencing the pathogenicity, transmissibility, and immune responses following host infection (7). Therefore, understanding the clinical characteristics of emerging variants of the coronavirus and investigating the evolutionary patterns of viral mutations can offer novel insights into predicting the emergence of future variant strains and identifying crucial neutralizing antibody binding sites. This is crucial for controlling the spread of the disease and preventing future outbreaks.

Porcine epidemic diarrhea virus (PEDV), belonging to the genus Alphacoronavirus, was first reported in the 1970s in the United Kingdom and Belgium (8). Before 2010, the PEDV G1a classic subgroup in China was limited to sporadic and regional outbreaks, attributed to the extensive use of PEDV CV777 inactivated and attenuated vaccines, which effectively contained infection rates (9). However, in October 2010, a highly pathogenic PEDV G2a variant strain emerged in southern Chinese provinces, rapidly spreading nationwide. Even pig farms immunized with the classical strain vaccine experienced a 100% mortality rate in newborn piglets (10). In 2013, the United States also faced a highly pathogenic PEDV strain classified as G2b, causing a 10% mortality rate in the national pig population and spreading rapidly to other countries worldwide, leading to significant losses in the global swine industry (11). Following the outbreak of the G2b strain, the United States witnessed the emergence of a new variant strain characterized by extensive nucleotide insertions and deletions in the S gene, named the S-INDEL strain (12). Despite the widespread use of commercial vaccines against the G1 and G2 subgroups of PEDV in China, recent epidemiological studies indicate that the prevalence and infection rates of PEDV have remained persistently high in recent years, posing a significant challenge to the country’s swine industry (13).

Many studies revealed that mutations in the S protein are the primary drivers of changes in the virulence and infectivity of PEDV. Currently, at least five neutralizing epitope regions have been identified within the S protein, including the N-terminal domain S1^0^ (NTD/S1^0^), the core neutralizing epitope region COE, the SS2 epitope region of the S1^D^ structural domain, the SS6 epitope region, and the 2C10 epitope region at the C-terminus (14–16). Through comparative analysis of the amino acid sequences of S proteins from representative strains of different subtypes, it was observed that the genetic variability within strains of the same subtype is low, while significant differences exist in the S gene among strains of different subtypes. The region with the greatest disparity in S protein lies in the N-terminal 1–400aa, encompassing the entire S1^0^ neutralizing epitope region (17). Through comparative analysis of the amino acid sequences of the S proteins of vaccine strains CV777, ZJ08, and various reference strains of different subtypes, it was observed that the neutralizing epitope regions in SS2 and 2C10 were the most conserved, while the neutralizing epitope regions in SS6 and COE exhibited more noticeable variability. In comparison to G1b vaccine strains like CV777 and ZJ08, the SS6 epitope region showed substitutions at serine residues L763S and D765S. The amino acid variations in the core neutralizing epitope region COE were found to be complex, with G2c and S-INDEL strains sharing amino acid replacements at positions 516, 548, 593, and 632 in the COE epitope region, and G2a and G2b strains displaying varying degrees of amino acid substitutions (17). Research has confirmed that mutations in the S gene sequence are the main drivers of PEDV virulence changes, although the specific key amino acid mutations in the S gene that play crucial roles remain unclear.

This study aims to systematically screen mutant strains that evade various neutralizing antibodies under the conditions of antibody pressure. It seeks to identify the crucial amino acid residues targeted by neutralizing antibodies, assess the impact of alterations in these residues on virus properties, and validate their role in the pathogenicity of PEDV and antibody neutralization by the S protein through animal trials. It is anticipated that the study’s results will provide new scientific knowledge for enhancing the efficacy and safety of vaccines. This has significant theoretical and practical implications for the monitoring and assessment of the potential emergence of resistance to antibody-based therapies and vaccines during their implementation.

## MATERIALS AND METHODS

### Virus and cells

The PEDV strain AH2012/12 (GenBank accession: KU646831) was isolated from the intestine of piglets and subsequently stored in our laboratory. The strain was cultured in Dulbecco’s modified Eagle’s medium (DMEM, Gibco, USA) containing 5 µg/mL trypsin (Sigma) and 37.5 µg/mL pancreatin (Sigma) (18). Vero cells (ATCC No. CCL-81) cells were cultured in DMEM supplemented with 10% fetal bovine serum (FBS) in a 5% CO_2_ atmosphere at 37°C.

### Generation of polyclonal antibody (pAb) against PEDV

The purified PEDV AH2012/12 particles were inactivated with a 0.1% formaldehyde solution for 2 h at 37°C and then emulsified with Fuchs complete adjuvant (Sigma). One 4-week-old specific pathogen-free (SPF) grade piglet was immunized with the mixture. The piglet received four intramuscular injections of PEDV, 4 weeks apart. Porcine serum was collected via the antecubital vein five days after the last injection, treated at 37°C for 2 h, and centrifuged at 12,000 rpm for 5 min. The serum was subsequently stored at −20°C.

### Generation of the pAb-escape virus by serially passaged

First, the PEDV AH2012/12 was mixed with pAb (1:10 dilution) and incubated at 37°C for 1 h. Then, the monolayers of Vero cells in a 24-well plate were inoculated with the mixture. After incubation at 37°C for 2 h, the mixture was discarded, and the cells were washed three times with DMEM. The Vero cells then continued to be cultured in the presence of pAb (1:10 dilution) supplemented with 5 µg/mL trypsin and 37.5 µg/mL pancreatin. Meanwhile, a control group of Vero cells was infected with PEDV AH2012/12 in the absence of pAb. Upon observation of cytopathic effect (CPE), the viruses were harvested and subjected to pAb-pressure passaging in the subsequent generation. If no CPE was observed after 48 h, the cells were washed twice and replaced with DMEM solution supplemented with 5 µg/mL trypsin and 37.5 µg/mL pancreatin without the pAb until CPE was observed. The S gene of the AH2012/12 was sequenced every 10 passages. The serial passaging of the pAb-pressure virus was terminated when the CPE was observed in both groups simultaneously.

### Generation of the recombinant PEDVs

The recombinant PEDV strains were constructed using CRISPR/Cas9 technology, as previously described (19). Three pairs of sgRNAs targeting the PEDV S gene were generated by overlapping polymerase chain reaction (PCR) and *in vitro* transcription, respectively. PCR amplification was conducted using the sgRNA-F/sgRNA-R primer pair and the constant reverse primer Scaffold oligo. The reaction was initiated at 98°C for 3 min and proceeded through 34 cycles of 98°C for 30 s, 55°C for 30 s, and 72°C for 30 s. Subsequently, the reaction was maintained at 72°C for 5 min using the SuperFiGreen PCR Master Mix (Invitrogen). Then, the PCR amplicons were purified using a DNA purification kit (Omega Bio-tek) and employed as the templates for sgRNA transcription. The transcription of sgRNA was conducted in a 20 µL mixture comprising 2 µL of 10× transcription buffer, 2 mM NTPs, 40 units of RNase inhibitor (TaKaRa), 1 µL DTT, and 100 units of T7 RNA polymerase (NEB) at 37°C overnight. The resulting sgRNAs were purified using phenol-chloroform and eluted in RNase-free water. Subsequently, the recombinant BAC plasmid carrying the AH2012/12 genome was then cleaved *in vitro* using CRISPR/Cas9. After purification of the digested recombinant BAC plasmid, homologous recombination fragments were ligated into the BAC plasmid by homologous recombination using the Infusion Cloning Kit (TaKaRa). The presence of the desired sequence in the positive clones was confirmed by PCR and Sanger sequencing (Nanjing Qingke Biotechnology Co., LTD, China). The primer sequences employed in this study are presented in Table S1.

### Viral plaque assay

The 10-fold serial dilutions of PEDV strains were incubated with six-well plates filled with monolayers of Vero cells for 1.5 h at 37°C. Subsequently, the cells were washed twice with DMEM to remove the unabsorbed virus and then covered with 2 mL of DMEM containing 1.5% methylcellulose, 5 µg/mL of trypsin, and 37.5 µg/mL of trypsin. After incubation at 37°C for 48 h, the cells were fixed with 4% paraformaldehyde and stained with 0.1% crystal violet.

### Viral growth kinetics analysis

Vero cells were inoculated in 24-well plates with the corresponding PEDV at a 0.1 MOI for 1.5 h at 37°C. After incubation, the cells were washed twice with DMEM to discard the viral supernatant. Then, 500 µL of DMEM medium, supplemented with 5 µg/mL trypsin and 37.5 µg/mL pancreatin, was added to each well. The cell supernatants were collected at various time points. The virus titer was performed using the 50% tissue culture infectious dose (TCID_50_) method.

### Immunofluorescence assay (IFA)

Vero cells in a 48-well plate were infected with 0.1 MOI of the corresponding PEDV. After 24 h of infection, the supernatant was removed, and the cells were fixed with 4% paraformaldehyde for 15 min at room temperature. Then, the cells were fixed with a pre-cooled methanol solution for 10 min and blocked with 5% skimmed milk at 37°C for 1 h. Subsequently, the cells were washed three times with phosphate-buffered saline (PBS) and incubated with a mouse anti-PEDV N monoclonal antibody (1:500 dilution) for 1 h. Following three washes with PBS, the cells were incubated with a goat anti-mouse IgG antibody (1:2,000 dilution) coupled with FITC (Beyotime) for 1 h and 0.1% DAPI (Boster) for 15 min at room temperature. Finally, the cells were washed three times with PBS, after which the fluorescence distribution was observed under a fluorescence microscope (Nikon).

### Virus neutralization assay

To determine the neutralization ability of the pAb against the corresponding PEDV, a pAb neutralization assay was performed according to the method previously described by Song et al. (20). Briefly, the pAb was first inactivated at 56°C for 30 min, then serially diluted twofold and mixed with an equal volume of PEDV (200 TCID_50_/100 µL). The mixture was then incubated at 37°C for 1 h. After incubation, 10 µg/mL of trypsin was added. The pAb–virus mixture was then inoculated onto monolayers of Vero cells in a 96-well tissue culture plate. After incubation at 37°C for 2.5 h, the mixture was discarded, and the cells were washed three times with DMEM. Finally, intracellular CPE was observed for 3–5 days.

### RNA extraction and real-time quantitative PCR (RT-qPCR)

Total RNA was extracted from Vero cells infected with the corresponding PEDV, intestinal swabs, or tissue samples using the Total RNA Isolation Kit (Vazyme Biotech Co., Ltd.). RNA was then reverse transcribed into cDNA by HiScript II Q RT SuperMix (Vazyme Biotech Co., Ltd.). RT-qPCR was performed on an ABI QuantStudio 6 Flex RT-qPCR system (Applied Biosystems) using the SYBR Green PCR Master Mix (Applied Biosystems). For the Vero cells, the PEDV N mRNA expression levels of mRNA were normalized to that of GAPDH and calculated using the 2-^ΔΔ^Ct method. For the fecal swab or intestinal tissue samples, viral RNA content was detected by absolute fluorescence quantitative PCR. The following PEDV-N gene primer and probe sequences were used: sense, 5′-CTGTTGTTGCCATTGCCACGA3', antisense: 5′-GTCTGAAAAGCCAATCATTC-3′, and probe 5′-TTGCCTCTGTTGTTACTC-3′.

### pAb inhibited the adsorption of PEDV

To determine the effect of pAb on virus adsorption, pAb (1:10 dilution) was incubated with PEDV for 1 h at 37°C. Then, the Vero cells were pre-cooled at 4°C for 30 min and incubated with the mixture for 1 h at 4°C to allow viral attachment without internalization. Cells were washed three times with ice-cold PBS to remove unbound virus. Total RNA was extracted from some cells to assess the effect of pAb on PEDV adsorption. The remaining cells were fixed and blocked to perform an IFA, and the fluorescence distribution was observed under confocal fluorescence microscopy (Zeiss). The green fluorescence intensity was quantified using Image J software.

### Immunoblot analysis

Vero cells in six-well plates were infected or mock-infected with PEDV AH2012 at 0.1 MOI. After 24 h post-infection (hpi), cells were washed with cold PBS and radioimmunoprecipitation assay (RIPA) buffer (Sigma) containing protease and phosphatase inhibitors (Roche). For immunoblot analysis, lysates were boiled at 100°C for 10 min and then separated by 10% SDS-PAGE. Proteins from SDS-PAGE were electroblotted onto a polyvinylidene difluoride membrane (Millipore). After blocking with PBS containing 5% skim milk, the membranes were incubated with pAb at 37°C for 1 h. After three washes in Tris-buffered saline with Tween (TBST), the membranes were incubated with horseradish peroxidase (HRP)-conjugated secondary antibodies for 1 h at room temperature. Finally, the membranes were detected with Western ECL substrate using the Bio-Rad ChemiDoc Imaging System for detection.

### Evaluation of the pathogenicity of recombinant PEDVs

A total of 15 5-day-old piglets, which were negative for the PEDV antigen and antibody, were randomly divided into three groups (five piglets per group): rAH2012/12 group, rA1273P group, and control group. Each group of piglets was challenged orally with a designated virus at 1 × 10^4.5^ TCID_50_. Following the challenge, daily clinical observations were made, and rectal swabs were taken to determine virus shedding by RT-qPCR. When piglets showed signs of death or at the end of the study, they were euthanized, and their small intestines were fixed in a 10% formaldehyde solution for HE staining and immunohistochemical analysis.

### Challenging the piglets after immunization with the AH2012/12 vaccine

A total of 25 5-day-old piglets, which were negative for the PEDV antigen and antibody, were randomly divided into five groups (five piglets per group) and kept in separate rooms. Piglets in the immunized group were injected intramuscularly with an inactivated PEDV vaccine, and piglets in the control group were injected intramuscularly with PBS. At 14 days post-vaccination (dpv), piglets in the immunized group received a booster vaccination. Serum was collected from piglets before vaccination and at 14 and 24 dpv. Virus challenge experiments were performed at 24 dpv. Piglets were orally challenged with the 2.0 × 10^6^ TCID_50_ PEDV strain. Daily clinical observations were made after the challenge, and rectal swabs were collected to determine virus shedding by RT-qPCR. When piglets showed signs of death or at the end of the study, they were euthanized, and their small intestines were fixed in a 10% formaldehyde solution for HE staining and immunohistochemical analysis.

### Modeling of PEDV S protein

The structure of heptad repeat (HR) regions and S protein of the parental strain and rA1273P strain was analyzed by AlphaFold3. AlphaFold3 was run through its public web server (https://www.alphafoldserver.com) (21). PyMOL software was used to visualize and compare the modeled tertiary structures.

### Statistical analysis

All data were expressed as mean ± standard deviation (SD) and were statistically analyzed using GraphPad Prism software 8 (GraphPad Software, Inc.). The two-tailed unpaired *t*-test was used for comparison between the two groups, and the Bonferroni multiple comparison test was used for comparison between multiple groups. Statistical significance is indicated by *, *P* < 0.05; **, *P* < 0.01; ***, *P* < 0.001.

## RESULTS

### PEDV S exhibits multipoint mutation under antibody pressure

**Fig 1:**
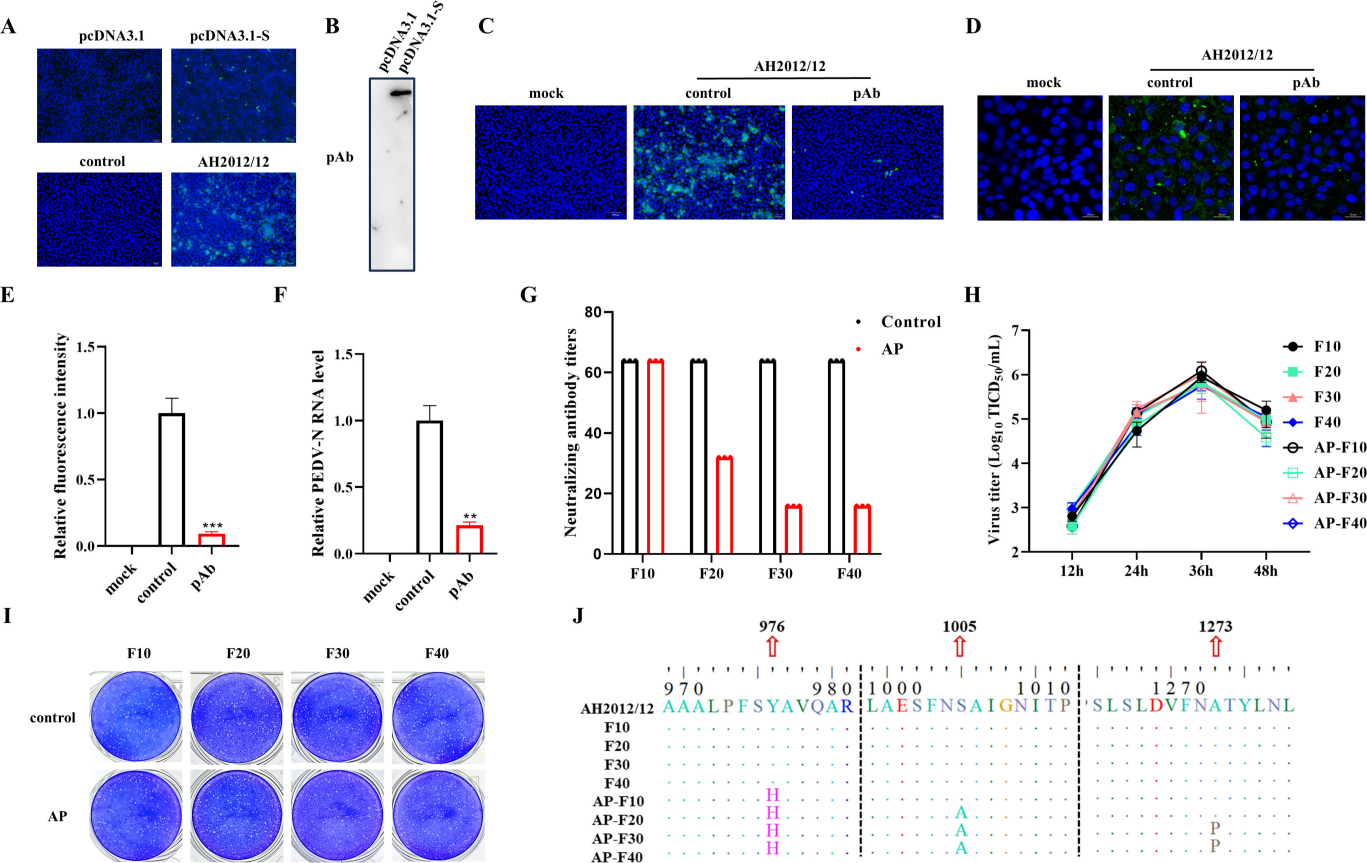
PEDV S exhibits multipoint mutation under antibody pressure. (**A**) The PEDV pAb was identified by IFA. (**B**) The PEDV pAb was identified by Western blotting. (**C**) The neutralizing effect of pAb on the virus was detected by IFA. (**D**) IFA results showing inhibition of virus adsorption by pAb. (**E**) The green fluorescence intensity was quantified using Image J software. (**F**) RT-qPCR results indicating inhibition of virus adsorption by pAb. (**G**) Neutralization effects of pAb on different viruses. (**H**) The growth curve of the continuous passage of viruses. (**I**) Plaque assay results of the continuous passage of viruses. (**J**) Sequencing results of PEDV S gene after different passages under pressure conditions.

In order to imitate the evolution of the PEDV under the influence of therapeutic antibody pressure, a hyperimmune serum (pAb) was prepared. The IFA demonstrated the specific binding of the pAb to the overexpressed S protein and PEDV-infected cells (Fig. 1A). Western blotting also demonstrated specific binding of the pAb to the overexpressed S protein (Fig. 1B). In order to determine the neutralizing capacity of pAb against PEDV, the virus neutralization experiments were conducted. The results of the IFA demonstrate that the pAb can effectively neutralize viral infections and reduce the number of infected cells (Fig. 1C). Furthermore, IFA and RT-qPCR results indicated that the pAb significantly inhibited PEDV adsorption (Fig. 1D through F). To identify the key sites for pAb, PEDV was continuously passaged under antibody pressure to obtain the pAb-escape mutant virus. The neutralization experiment results demonstrate a gradual reduction in neutralizing antibody titers against the 20th, 30th, and 40th generations of the virus under pAb pressure, while the 10th virus has no change (Fig. 1G). The growth kinetics and plaque assay showed that there was no significant difference between the pAb pressure virus and the wild-type strains (Fig. 1H and I). Sequencing of the original virus at different passages and the virus S gene under pressure revealed a Y to H mutation at amino acid position 976 in the 10th generation virus, an S to A mutation at position 1,005 in the 20th generation virus, and an A to *P* mutation at position 1,273 in the 30th generation virus (Fig. 1J). These mutations at key amino acid positions under neutralizing pressure suggest potential immune escape mechanisms of the coronavirus from host immunity.

### Construction and characteristics of recombinant PEDV strains

**Fig 2:**
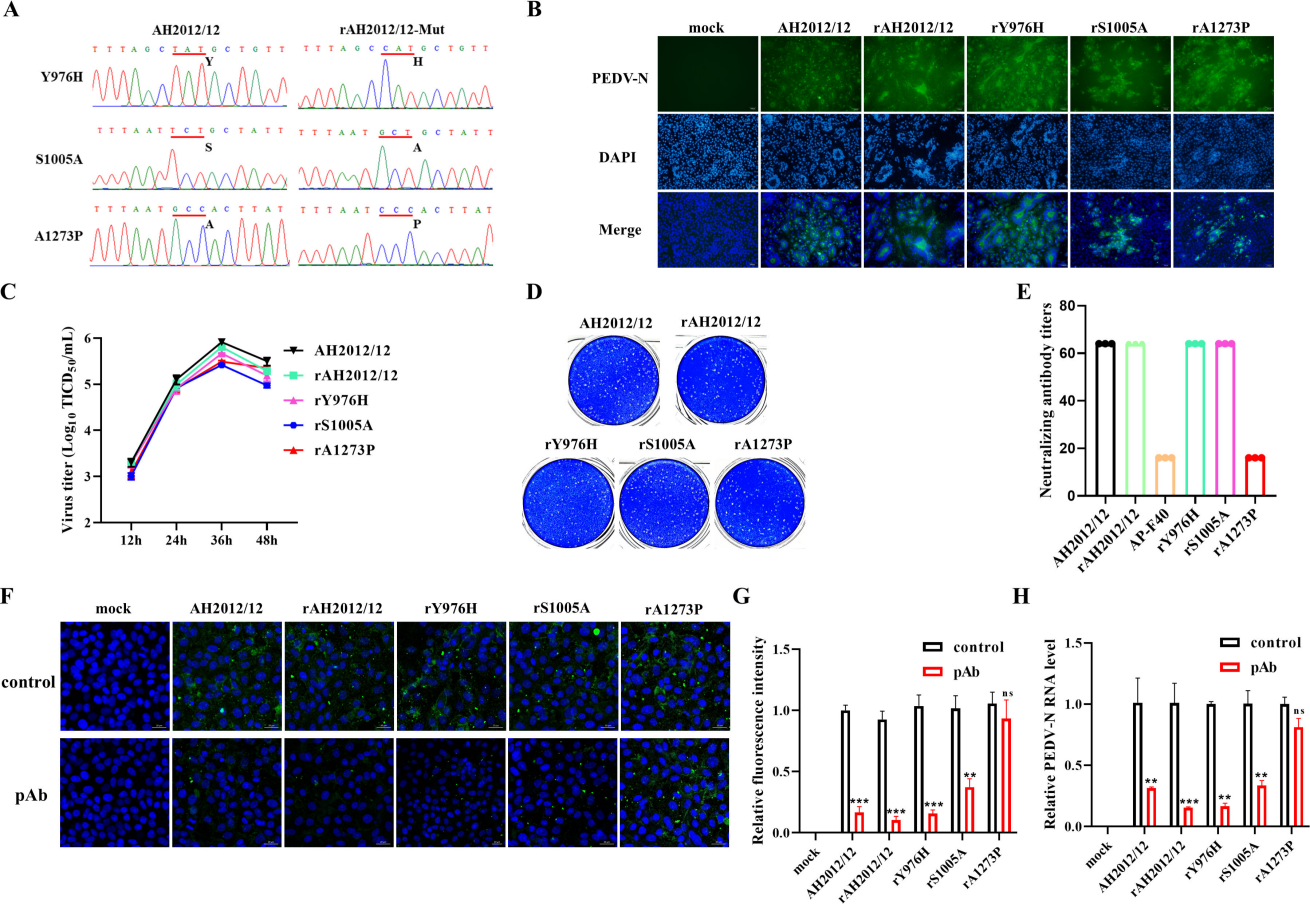
Construction and characteristics of recombinant PEDV strains. (**A**) Successful construction of three recombinant viruses. (**B**) Identification of the recombinant viruses by IFA. (**C**) The growth curve of the recombinant viruses. (**D**) Plaque assay results of the recombinant virus. (**E**) Neutralizing ability of pAb against various recombinant viruses. (**F**) IFA results of pAb inhibition of multiple recombinant virus invasions. (**G**) The green fluorescence intensity was quantified using Image J software. (**H**) RT-qPCR results of pAb inhibition of multiple recombinant virus invasions.

To validate the significance of three amino acid residues, we successfully generated three recombinant strains, namely rY976H, rS1005A, and rA1273P, using a reverse genetic system, with the mutation sites confirmed by sequencing (Fig. 2A). To evaluate the characteristics of the mutated strains, pathogenic observation and growth curve were conducted. The results of the IFA assay demonstrated that the recombinant strains were capable of infecting cells and induced cytopathic effects similar to the wild-type strains (Fig. 2B). The growth kinetics of the viruses indicated a comparable growth trend between the recombinant strains and the wild-type strains (Fig. 2C). Additionally, the results of the plaque assay show that there was no difference in plaque morphology between the three recombinant viruses and the wild-type strains (Fig. 2D). To further confirm the key mutation that is able to evade antibody pressure, the neutralizing antibody titers against various strains were tested. The neutralization assays utilizing pAb demonstrated a reduction in neutralizing antibody titers against the 40th passage virus and the recombinant virus rA1273P (Fig. 2E). Furthermore, the results of the IFA and RT-qPCR experiments indicated that the recombinant virus rA1273P exhibited a notable capacity to evade neutralization by serum antibodies, in comparison to the other strains (Fig. 2F through H). These findings revealed the significance of the A to *P* mutation at position 1273 of the PEDV S gene.

### Virulence evaluation of the recombinant viruses

**Fig 3:**
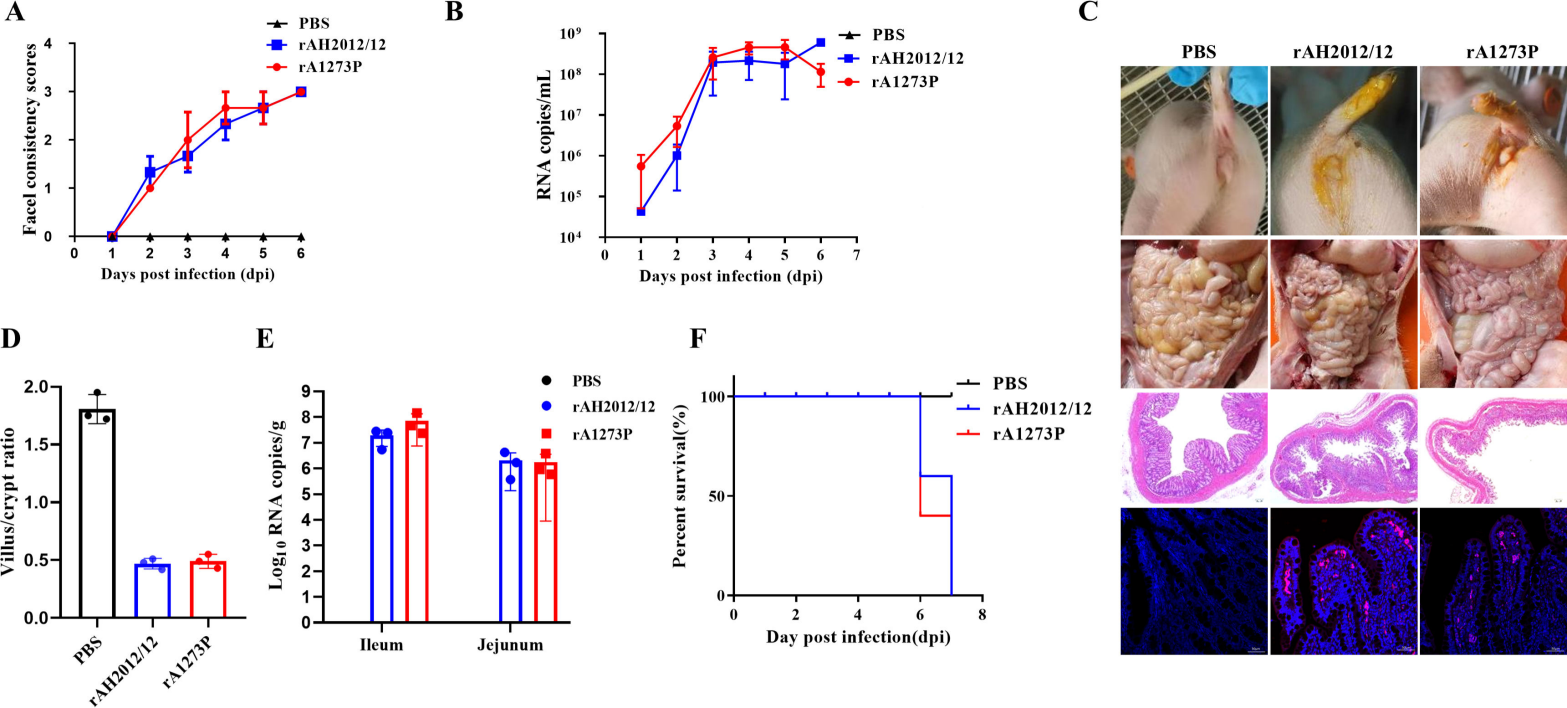
Virulence evaluation of the recombinant viruses. (**A**) Statistics on fecal diarrhea in piglets caused by wild-type and the mutant virus. Diarrhea scores were 0, solid; 1, pasty; 2, semiliquid; 3, liquid. (**B**) Daily quantification of viral shedding in rectal swabs after oral administration of PEDV. (**C**) Piglet diarrhea status, anatomical diagrams, HE staining, and IFA results. (**D**) Statistical analysis of the villus-to-crypt ratio in piglet intestines. (**E**) Viral loads in the ileal and jejunal segments of piglets. (**F**) The survival rates of piglets.

A challenge test was undertaken on 5-day-old piglets to deepen the evaluation of the virulence of the recombinant viruses. The severity of diarrhea caused by the recombinant virus rA1273P was comparable to that induced by the wild-type virus, as indicated by the fecal scores (Fig. 3A). The results of fecal shedding demonstrated a comparable shedding pattern between the recombinant and wild-type viruses, with peak shedding occurring on the third-day post-infection (Fig. 3B). The clinical records revealed that both the recombinant and wild-type viruses resulted in severe diarrhea, accompanied by intestinal distension, thinning, and transparency of the intestinal wall (Fig. 3C). Additionally, histological analysis revealed the presence of intestinal villi atrophy and disruption were observed, accompanied by a statistically significant reduction in the height-to-crypt depth ratio in the challenged group. No notable difference was observed between the recombinant and wild-type viruses (Fig. 3D). Immunofluorescence staining of the villi provided additional confirmation of viral infection in the intestinal villi (Fig. 3C). Moreover, there was no significant difference in viral load in jejunum and ileum between the two challenge groups (Fig. 3E). The survival curve showed that all piglets in both the challenge and wild-type groups succumbed on the 7th day (Fig. 3F). These findings demonstrated that the mutant rA1273P strain has comparable pathogenicity to the wild-type strains.

### Recombinant viruses escape neutralized antibody *in vitro*

**Fig 4:**
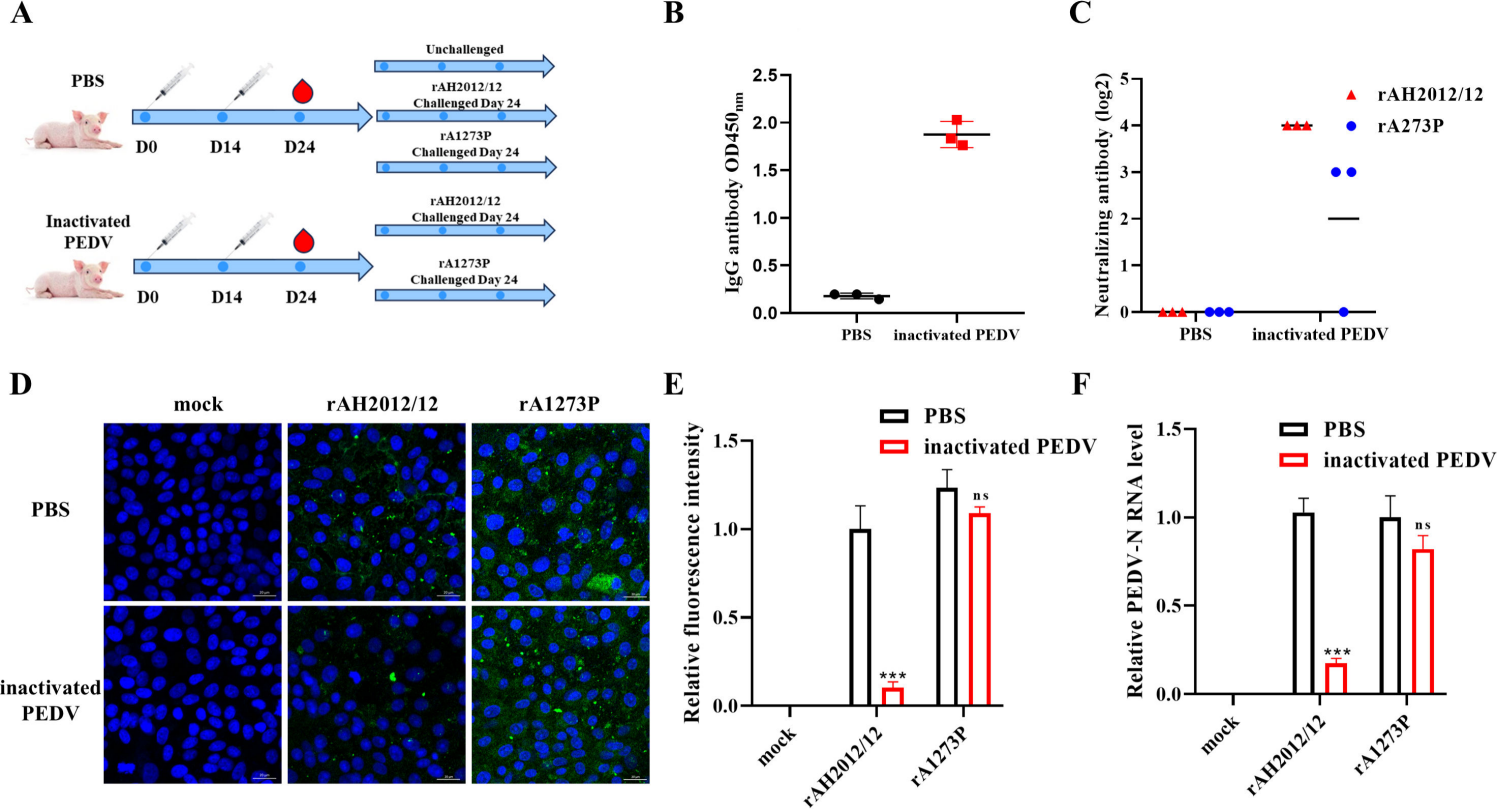
Recombinant viruses escape neutralized antibody *in vitro.* (**A**) Schematic diagram of the immunization and challenge process in animal experiments. (**B**) Detection of IgG antibody levels in pigs before challenge following immunization. (**C**) The neutralizing ability of serum after immunization against recombinant and parental viruses. (**D**) IFA results are obtained to measure the adsorption capacity of serum after immunization against recombinant and parental viruses. (**E**) The green fluorescence intensity was quantified using Image J software. (**F**) RT-qPCR analysis is conducted to determine the adsorption capacity of serum against recombinant and parental viruses.

Subsequent to the confirmation of the comparable pathogenicity of the recombinant virus to the wild-type virus, further experiments were conducted to verify its capability to evade antibodies *in vitro*. In order to substantiate the potential immune evasion of the recombinant virus in pigs, 5-day-old piglets underwent two rounds of immunization with inactivated AH2012/12 PEDV or PBS, as outlined in Fig. 4A. Additionally, five piglets were unvaccinated and unchallenged as the controls. After 24 days post-immunization, the piglets were challenged. Prior to the challenge, the results of the enzyme-linked immunosorbent assay (ELISA) demonstrated significantly elevated levels of IgG antibodies in the serum of the immunized group compared with the PBS group (Fig. 4B). The lack of protective efficacy of serum against the mutant rA1273P strain was confirmed through *in vitro* validation using neutralizing antibody levels, IFA, and RT-qPCR results. Conversely, the post-immunization serum demonstrated robust protective capacity against the wild-type rAH2012 virus (Fig. 4C through F).

### Recombinant viruses escape neutralized antibody *in vivo*

**Fig 5:**
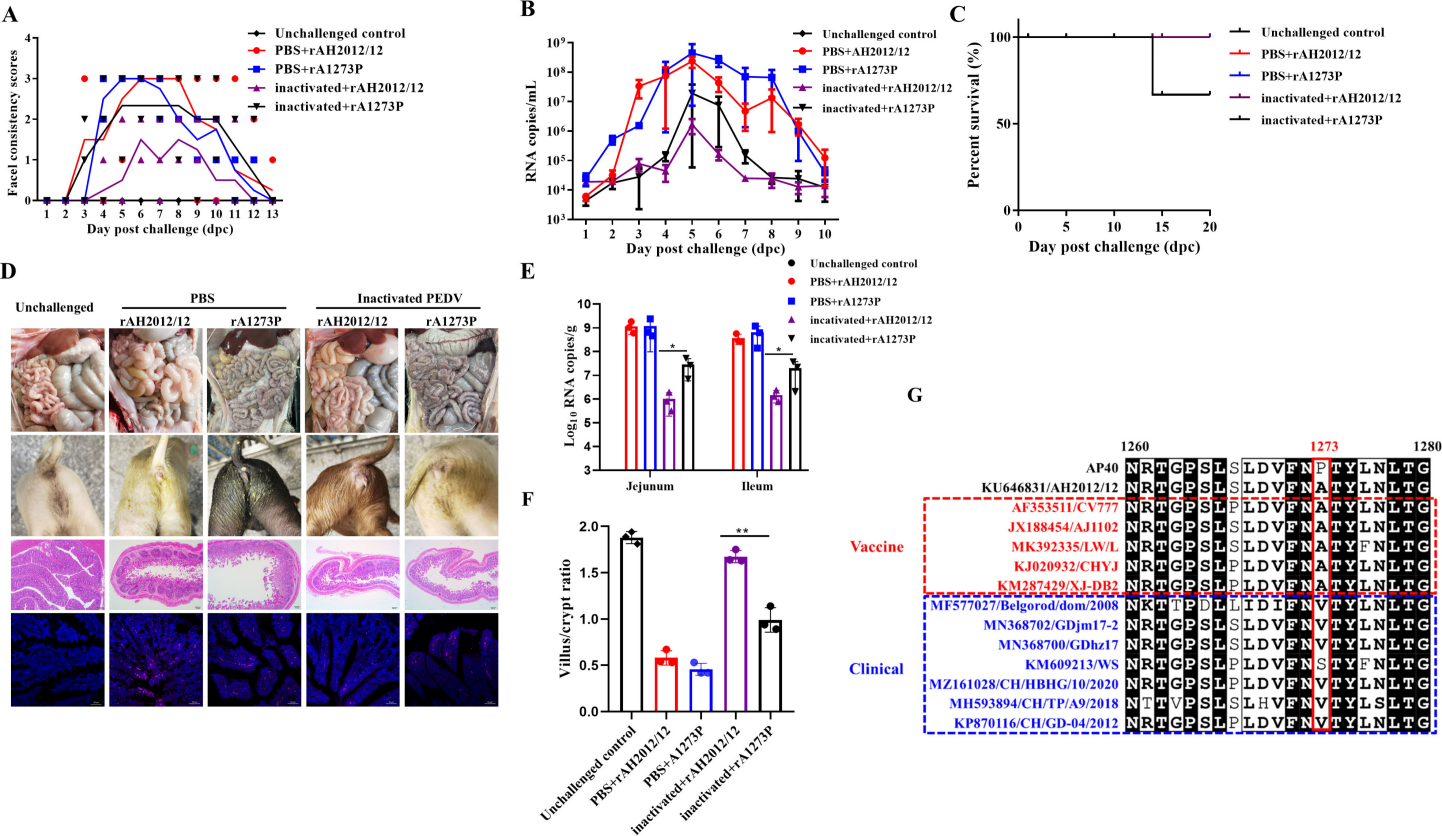
Recombinant viruses escape neutralized antibody *in vivo.* (**A**) Diarrhea scores of animals in different groups. Diarrhea scores were 0, solid; 1, pasty; 2, semiliquid; 3, liquid. (**B**) Daily quantification of viral shedding in rectal swabs after oral administration of PEDV. (**C**) Survival rates among animals in different groups. (**D**) Piglet diarrhea status, anatomical diagrams, HE staining, and IFA results. (**E**) Viral loads in the ileal and jejunal segments of piglets. (**F**) Statistical analysis of the villus-to-crypt ratio in piglet intestines. (**G**) Sequence alignment results comparing the 1,273th amino acids of vaccine strains with clinically isolated viral strains.

In order to further assess the potential escape of neutralizing antibodies by the recombinant virus, the clinical symptoms in piglets post-challenge were monitored. The diarrhea scores indicated that the PBS group exhibited more severe diarrhea than the immunized group, with the rA1273P strain causing more severe diarrhea compared with the parental strain in the immunized group (Fig. 5A). The unvaccinated and unchallenged piglets exhibited no signs of diarrhea throughout the experiment. The results of the viral shedding analysis of the anal swabs indicated that the PBS group exhibited higher levels of shedding than the immunized group, with the rA1273P strain demonstrating slightly elevated levels compared with the parental strain in the immunized group but lower than the PBS group (Fig. 5B). A survival curve analysis revealed that only one mortality in the group infected with the rA1273P strain post-immunization, with no significant difference observed (Fig. 5C). The clinical manifestations, including gross pathology, histological sections, and immunofluorescence results, demonstrated severe diarrhea in the piglets from the PBS group, with villous atrophy, rupture, and viral infection evident (Fig. 5D). In the immunized group, the rA1273P strain was observed to cause more severe diarrhea, damage to the villi, and a higher viral load than the parental strain, while no significant pathological changes were observed in the unchallenged piglets. Moreover, viral loads in the jejunum and ileum were significantly higher in the immunized group infected with the recombinant virus compared to the parental strain (Fig. 5E). The statistical analysis of histopathological sections also revealed a reduction in villus-to-crypt ratios and an increase in the severity of villous damage with the recombinant virus strain (Fig. 5F). Furthermore, a comparative analysis of the S protein amino acid position 1,273 among over a thousand PEDV strains in the NCBI database revealed that all commercial vaccine strains exhibited an amino acid A at position 1,273, while seven clinical strains exhibited variations (Fig. 5G). These findings suggest that the use of vaccines and therapeutic antibodies may impact the escape and mutation of PEDV, with the specific mechanisms of variation requiring further exploration.

### The A1273P mutation in the S protein does not alter the overall architecture

**Fig 6:**
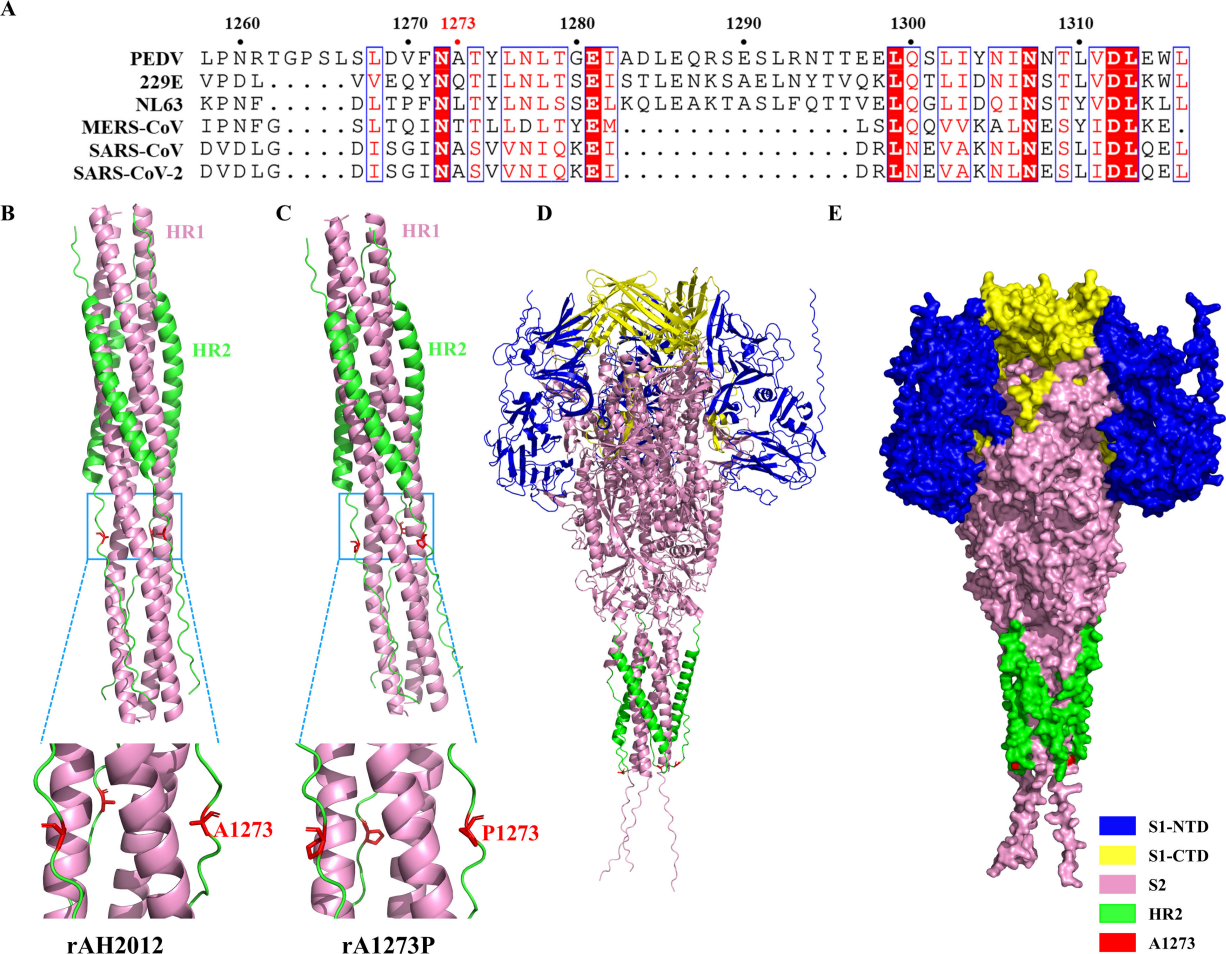
The A1273P mutation in the S protein does not alter the overall architecture. (**A**) Sequence alignment of the HR2 region of PEDV (AMZ01557), HCoV-229E (AAK32191), HCoV-NL63 (AAS58177), MERS-CoV (AHC74098), SARS-CoV (AAP13441), and SARS-CoV-2 (YP_009724390). The structural features of local and global HR regions (HR1: pink; HR2: green; 1,273th: red) protein predicted by AlphaFold3 for the parental strain (**B**) and rA1273P strain (**C**). The cartoon (**D**) and surface (**E**) of S protein were labeled in the sequence view picture of PEDV S, predicted by AlphaFold3 (S1-NTD: blue; S1-CTD: yellow; S2: pink; HR2: green; A1273: red).

The S glycoprotein of PEDV comprises two subunits, S1 and S2. The N-terminal S1 subunit mediates receptor binding and serves as the primary target for neutralizing antibodies (22). In contrast, S2 is responsible for virus–cell fusion and consists of several key domains: the protease cleavage site S2′, the fusion peptide (FP), heptad repeat 1 (HR1), heptad repeat 2 (HR2), transmembrane domain (TM), and intravirion tail (IV) (23). By comparing with the sequences of other coronaviruses, the 1,273th amino acid was found to be located in the HR2 region (Fig. 6A) (24). Notably, the HR regions are highly conserved motifs in viral glycoproteins that assemble into a six-helical bundle structure, which is essential for mediating membrane fusion during viral entry (25). Interestingly, recent studies have revealed that the HR regions also can elicit the production of neutralizing antibodies, further highlighting their potential significance in viral pathogenesis and immune response (24, 26).

Despite extensive research on the structural architecture of the PEDV S protein, the characterization of its HR regions remains insufficiently explored (22, 27, 28,). To investigate the impact of the A1273P mutation on the global architecture of the prefusion HR1HR2 bundle and the trimer structure of the S protein, we employed AlphaFold3 for further analysis (21). The results show that the A1273P mutation is located in the side-chain of HR2 and relatively far from the six-helix-bundle region (Fig. 6B and C). The mutation of A1273P at the side-chain position does not disrupt the overall structure of the HR1HR2 protein bundle and the trimer structure of the S protein consistent with previous research in severe acute respiratory syndrome coronavirus 2 (SARS-CoV-2) (Fig. 6B through D) (29). Based on these insights, we propose a mechanistic hypothesis wherein the pAb specifically target the HR regions, thereby inhibiting the HR-mediated viral membrane fusion process. Importantly, the A1273P mutation in the S protein appears to abolish this antibody-binding capacity, due to proline’s cyclic side chain-induced conformational changes in the protein, thereby enabling the virus to evade neutralization by pAb.

## DISCUSSION

The findings of this study demonstrate that under conditions of pAb immunity pressure, the PEDV S protein undergoes a series of point mutations. Specifically, the mutation of the amino acid at position 1,273 of the S protein from A to *P* enables the virus to evade neutralization by pAb immunity. Furthermore, animal experiments have demonstrated that the A1273P mutation of the S protein in the recombinant strain does not influence the viral virulence but allows evasion of antibody pressure under both *in vitro* and *in vivo* conditions, resulting in successful infection and pathogenesis in piglets.

The PEDV S protein is structurally segmented into S1 and S2 domains, with the S1 domain consisting of four core subdomains: S1^0^, S1^A^, S1^B^, and S1^CD^. The S1^B^ subdomain contains the primary receptor binding domain (RBD), which is located at amino acids 510–640 and binds to aminopeptidase N (APN) (30). Within the S1^A^ and S1^CD^ subdomains lie neutralizing epitopes, with five specific regions identified at amino acids 499–638, 636–789, 748–755, 746–771, and 1368–1374 (15, 31, 32). It has been demonstrated in previous studies that serial passaging of the virus *in vitro* can effectively reduce viral virulence (33, 34). In our study, serial passaging of the virus for 40 generations under pAb conditions did not result in decreased pathogenicity in piglets following oral administration. This is primarily attributable to our use of a rescue virus with a single mutation site rather than the serial passage of PEDV. Furthermore, the number of *in vitro* passage cycles was relatively limited, and we exclusively sequenced the S gene without conducting sequencing for other genes across the entire genome. Previous research identified mutations associated with attenuation in passaged strains, such as S1A (D265A), RBD (F635R), and COE (T502I, T555S, and G600S) (35). Furthermore, sequencing analysis of the S gene revealed approximately 50 amino acid mutations in comparison to both the clinical strain and the vaccine strain CV777. As previously observed, the mutations in the S protein were primarily situated in the N-terminal structural domain. Specifically, there were 8–12 variable mutation sites in the COE epitope region, only one amino acid mutation site in the SS6 epitope region, and no mutations in the SS2 epitope region (36). Amino acid substitutions within these regions have been demonstrated to diminish the binding affinity, antigenicity, pathogenicity, and neutralizing activity of PEDV (37–39). Mutations observed after 40 passages were mainly located in the S2 region in our study, while mutations leading to attenuation were predominantly in the S1 region as reported. These findings suggest that the S1 region of PEDV may play a more critical role in virulence, while the functionality of the S2 region requires further exploration.

Generally, the S1 domain mediates viral entry and attachment and induces neutralizing antibodies, while the S2 domain is responsible for membrane fusion (40). However, Okda et al. successfully generated eight highly neutralizing monoclonal antibodies through immunization of mice with the whole virus. Among these, seven antibodies demonstrated specific binding to the S2 subunit (41). Additionally, Feng et al. have identified a specific neutralizing monoclonal antibody, which effectively neutralizes a range of PEDV strains, including genotypes 1 (CV777) and 2 (LNCT2) (42). The core sequence of the neutralizing epitope is conserved within amino acids 1,261 to 1,337. These collective findings provide compelling evidence that the S2 subunit possesses the capability to induce the production of neutralizing monoclonal antibodies, challenging the traditional paradigm of S1-centric neutralization.

In our study, we identified a novel mutation A1273P near the C-terminus of the S2 protein under pAb pressure, which represents a newly characterized key binding site for neutralizing antibodies. This 1,273th amino acid site is located in the HR2 region of the S protein. The A1273P mutation resides in HR2’s side-chain distant from the six-helix bundle predicted by AlphaFold3. This side-chain substitution preserves both HR1HR2 structural integrity and S protein trimer formation according to the prior SARS-CoV-2 research (29). Previous research has established that the HR regions are a highly conserved structure in viral glycoproteins responsible for viral fusion. Many studies have shown that exogenous soluble HR2 peptides can bind viral HR1, thereby blocking viral entry and replication such as severe acute respiratory syndrome coronavirus (SARS-CoV), Middle East respiratory syndrome coronavirus (MERS-CoV), and mouse hepatitis virus (MHV) (43–46). Zhao et al. demonstrated that HR peptide can inhibit PEDV infection, and HR2 peptide is the most effective inhibitor (24). Immunizing mice with HR2 peptide can induce neutralizing antibodies, and the serum can still inhibit PEDV infection by 50% after 1:64 dilution, which is consistent with our findings (29). The A1273P mutation in the S protein, which allows viral escape from pAb neutralization, may be due to conformational changes in the epitope’s spatial structure caused by the cyclic side chain of proline. The use of polyclonal antibody pressures in this experiment introduces more complex factors compared to monoclonal antibody pressure. While *in vitro* animal experiments demonstrated significant immune evasion due to mutations at this site, further investigations should employ different monoclonal antibodies to screen for key sites where the virus easily mutates to escape. Although some neutralizing epitopes have been identified for PEDV, the specific amino acid mutations critical for evading S protein neutralizing antibodies remain undisclosed. Further exploration of the neutralizing epitope sequences on the PEDV S protein is imperative to lay the theoretical groundwork for developing neutralizing therapeutic antibodies and highly effective protective vaccines, thus providing technical means for the development of potent PED prevention vaccines.

In addition, monitoring and predicting the direction of PEDV evolution are crucial for expedited control of its spread. An evolutionary analysis of PEDV strains has shown that the viral evolution rate has tripled following the implementation of vaccines. The analysis of 672 representative PEDV strains from different countries showed a regional preference in the evolution pattern of the G2 subgroup strains of PEDV, with South Korean strains evolving the fastest and Chinese strains exhibiting the highest recombination rate (47). These findings suggest that widespread vaccination against PEDV or immune pressure have accelerated the viral evolution. Therefore, studying viral evolution under immune pressure *in vitro* can predict the direction of viral evolution, enabling timely preparation of control measures to mitigate infections and economic losses caused by variant viruses. This not only provides a technical basis for predicting viral evolution but also offers crucial insights for exploring key neutralizing sites and designing vaccines.

In this study, we used artificial mutagenesis to induce mutations in PEDV, a member of the coronavirus genus, under antibody pressure, successfully obtained mutant strains that can successfully escape neutralizing antibodies induced by the parent strain, and discovered new neutralizing epitopes that had not been reported before. The research findings provide a deeper understanding of the immune escape of the coronavirus and prompt new intervention strategies for the prevention and treatment of coronavirus infection.

## Data Availability

All data underlying our findings are contained in the article and supplemental material.

## SUPPLEMENTAL MATERIAL

Table S1 jvi.02193-24-s0001.docx Primer sequences for the construction of recombinant virus.

ASM does not own the copyrights to Supplemental Material that may be linked to, or accessed through, an article. The authors have granted ASM a non-exclusive, world-wide license to publish the Supplemental Material files. Please contact the corresponding author directly for reuse.
