# Supplementary material for: Molecular characteristics of the immune escape of coronavirus PEDV under the pressure of vaccine immunity
Source: J Virol. 2025 Apr 16;99(5):e02193-24. doi: 10.1128/jvi.02193-24 (PMC12090811; doi:10.1128/jvi.02193-24)
Supplement: Table S1 — Primer sequences for the construction of recombinant virus. [file jvi.02193-24-s0001.docx]

| Primer | Sequence |
| --- | --- |
| SgRNA Y976H-F | TTCTAATACGACTCACTATAGGCAGCATTACAACTCAGCGCTGTTTTAGAGCTAGA |
| SgRNA Y976H-R | TTCTAATACGACTCACTATAGGAAATCGCAATCTCAGCGTTAGTTTTAGAGCTAGA |
| Y976H-F | AGTCAGCATTACAACTCAGC |
| Y976H-R | GCTAAAAGGCAATGCCGCTG |
| Y976H-M-F | CAGCGGCATTGCCTTTTAGCCATGCTGTTCAAGCGAGACTG |
| Y976H-M-R | CACCACCACAAAAACCATAA |
| SgRNA S1005A-F | TTCTAATACGACTCACTATAGGTATGCTTACTATTTCTGAAGGTTTTAGAGCTAGA |
| SgRNA S1005A-R | TTCTAATACGACTCACTATAGGTTTACACACAGTACTTGTACGTTTTAGAGCTAGA |
| S1005A-F | CTCTATGCTTACTATTTCTG |
| S1005A-R | ATTAAAAGACTCAGCAAGCA |
| S1005A-M-F | TGCTTGCTGAGTCTTTTAATGCTGCTATTGGTAATATAACT |
| S1005A-M-R | ATTTACAAAGTCACCCGGTA |
| SgRNA Y1273H-F | TTCTAATACGACTCACTATAGGAAATCGCAATCTCAGCGTTAGTTTTAGAGCTAGA |
| SgRNA Y1273H-R | TTCTAATACGACTCACTATAGGTTCGGATGCTGTCCAAGAGTGTTTTAGAGCTAGA |
| Y1273H-F | CAACTCTATGCTTACTATTTCTG |
| Y1273H-R | GGGATTAAAAACATCTAGAGAAA |
| Y1273H-M-F | TTTCTCTAGATGTTTTTAATCCCACTTATCTTAATCTTACTGGTGAAATTG |
| Y1273H-M-R | TTGGAACTACATTAAGCTCCAACT |

**Supporting information**

**Table S1:** Primer sequence for the construction of recombinant virus.
